# Ultracompact Graphene-Assisted Tunable Waveguide Couplers with High Directivity and Mode Selectivity

**DOI:** 10.1038/s41598-018-31555-7

**Published:** 2018-09-06

**Authors:** Yuan Meng, Futai Hu, Yijie Shen, Yuanmu Yang, Qirong Xiao, Xing Fu, Mali Gong

**Affiliations:** 1 State Key Laboratory of Precision Measurement Technology and Instruments, Department of Precision Instrument, Tsinghua University, Beijing, 100084 China; 2 State Key Laboratory of Tribology, Department of Mechanical Engineering, Tsinghua University, Beijing, 100084 China

## Abstract

Graphene distinguishes itself as a promising candidate for realizing tunable integrated photonic devices with high flexibility. We propose a set of ultracompact tunable on-chip waveguide couplers with mode-selectivity and polarization sensitivity around the telecom wavelength of 1.55 μm, under the configuration of graphene-laminated silicon waveguides patterned with gold nanoantennas. Versatile couplings can be achieved in a widely tunable fashion within a deep-subwavelength area (210 × 210 nm^2^), by marrying the advantages of tight field confinement in plasmonic antennas and the largely tunable carrier density of graphene. Incident light signals can be selectively coupled into different fundamental modes with good mode quality and high directionality exceeding 25 dB. Design scenarios for asymmetric couplings are presented, where the operation wavelength can be tuned across a 107-nm range around 1.55 mm by altering the chemical potential of graphene from 0 to 1.8 eV. Furthermore, the proposed schemes can be leveraged as mode-sensitive on-chip directional waveguide signal detectors with an extinction ratio over 10 dB. Our results provide a new paradigm upon graphene-assisted tunable integrated photonic applications.

## Introduction

Plasmonic nanoantennas attract tremendous research interest for their excellent capabilities to confine light in subwavelength volume^1^. Judicious design and arrangement of these scatterers provide unprecedented degree of freedom in the manipulation of electromagnetic field, giving rise to a plethora of emerging applications such as enhanced light emission and detection^2,3^, sensing and optical metasurfaces^4,5^. The investigation focus has been so far primarily centered around tailoring the propagation of light waves in free space^6^, while comparatively fewer attentions are devoted to their eminent potentials regarding guided waves in photonic waveguides^7,8^.

Meanwhile, photonic integrated circuits have been hailed as an appealing platform for optical information processing^9^, lab-on-a-chip systems and ultrafast chip-scale optical interconnects with low power dissipation^10,11^, holding the promise for revolutionizing conventional electronics and technologies. Practically, one may frequently require selectively in-coupling certain optical signals into certain guided mode with high directionality or out-coupling some specific wavelength channels out of the waveguide for processing or detection. Consequently, as an indispensable component that bridges connection between free-space light waves and guided signals in waveguides, compact directional couplers and waveguide detectors are of vital significance. However, conventional optical coupling components such as prisms and grating couplers are generally bulky^12,13^. The introduction of plasmonic antennas can largely miniaturize device footprint to facilitate on-chip coupling applications. Recently demonstrated nanoantennas-based couplers are mainly concentrated on the directional launching of surface plasmon polaritons or their operation wavelengths are generally fixed once the devices are fabricated^14–19^. Graphene was successfully leveraged in tunable metasurfaces working for free-space optics from near- to mid-infrared bands^20–24^, but the combination of graphene plasmonic antennas and photonic waveguides for the sake of tunable on-chip coupling applications still remains elusive.

Here we numerically demonstrate a set of ultracompact tunable on-chip waveguide couplers under the synergy of plasmonic nanoantennas and graphene-laminated silicon waveguides, possessing the capability of highly directional, operation wavelength largely tunable, mode-selective and polarization-sensitive coupling from free-space light waves into photonic waveguides around the telecom wavelength of *λ*_0_ = 1.55 μm. Although the unidirectional excitation of the surface plasmon polaritons in graphene has been previously demonstrated^25–27^, our proposals instead focus on the electrically tunable coupling and manipulation of guided waves in dielectric waveguides, which remains an important topic for photonic integrated circuits. The maximum spectral tunability range is 107 nm with respect to *λ*_0_ and the directivity parameter is greater than 25 dB, fulfilling the aforementioned design requirements^28^. Moreover, these configurations can also achieve mode-selective optical signal extraction from nanophotonic waveguides, leaving the signal channels with other propagation direction and polarization modes undisturbed. The detection wavelength can be swiftly tuned by applied voltages with an simulated extinction ratio greater than 10 dB, providing a helpful pathway to the applications of waveguide signal detection and sensing.

## Results

### Fundamentals and design principles

As a monolayer of carbon atoms arranged in a two-dimensional (2D) honeycomb lattice, graphene exhibits extremely high carrier mobility, strong light-matter interaction and wide electrostatic tunability^28,29^. Allying with a family of 2D materials, their exceptional attributes have found fertile soil in the realm of photonics and electronics^30^ with vastly growing number of scientific studies every year, such as broadband modulators and photodetectors^31–37^. By shifting the chemical potential *μ*_*c*_ of graphene, we can effectively control its optical attributes via electrical gating^38^.The complex permittivity of graphene undergoes dramatic variations when *μ*_*c*_ is tuned to block interband transitions^31–34^. It facilitates the way of utilizing graphene to achieve electrically tunable optical antennas^20–24^.

**Figure 1:**
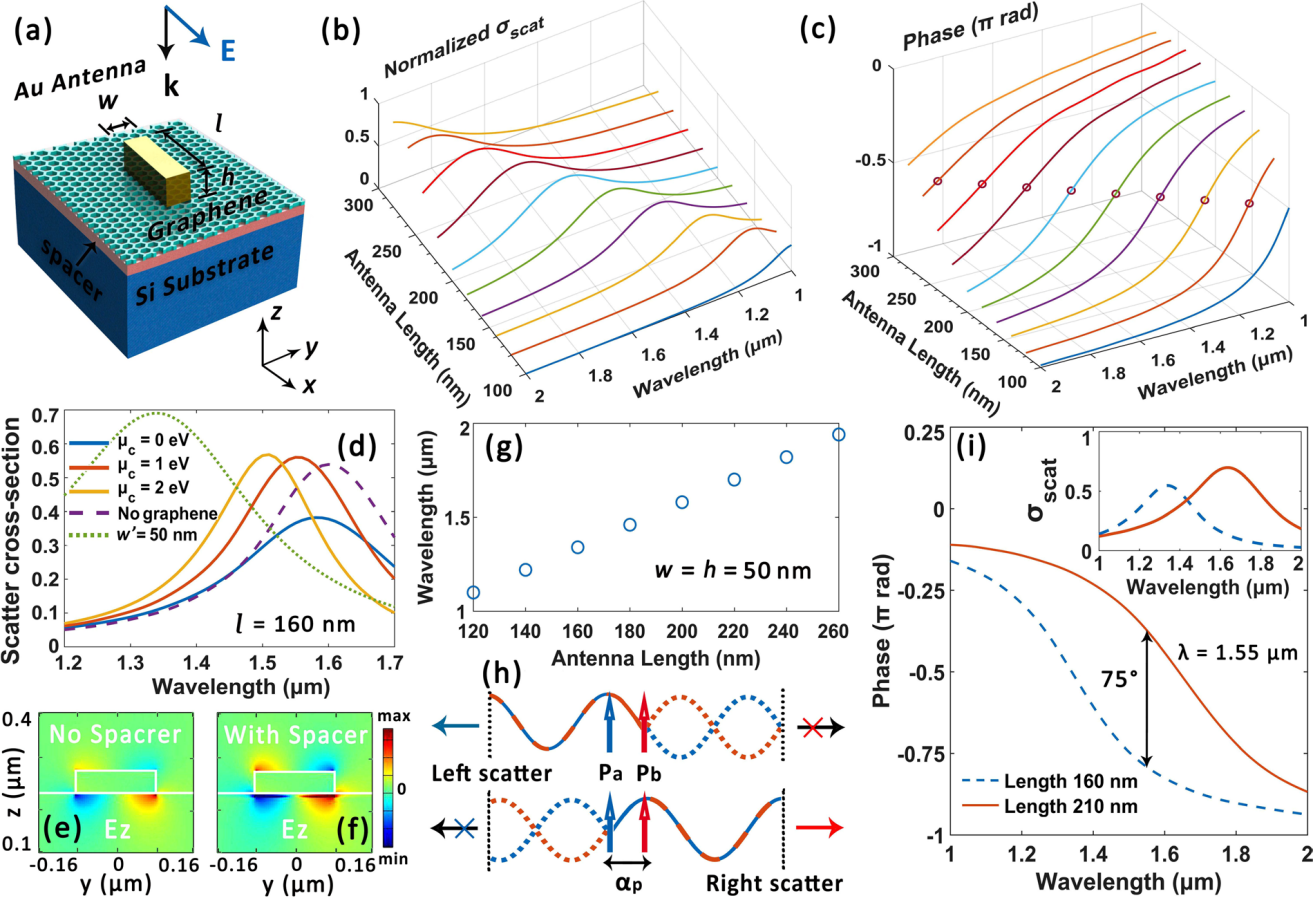
Antenna element design and analysis. (**a**) Simulation configuration sketch. (**b**) and (**c**) Normalized scattering cross-section *σ*_s*ca*_ and phase response of the gold antenna as a function of antenna length respectively (fiexed *w* = *h* = 50) nm. (**d**) Antenna tunability analysis. An exemplary *l* × *w* × *h* = 160 × 20 × 50 nm antenna is applied in the simulations for the solid and dashed lines. Antenna structure for green dotted line: *l* × *w*′ × *h* = 160 × 20 × 50 nm (*μ*_*c*_ = 0 eV) nm for comparison. (**e**) and (**f**) The distribution of electric field component *E*_*z*_ for a *l* × *w*′ × *h* = 160 × 20 × 50 nm gold antenna with the absence and presence of h-BN spacer layer respectively. (**g**) Resonant wavelength as a function of antenna length corresponding to (**b**). (**f**) The illustration of the electric dipole model for directional scattering. (**i**) Phase responses for two antenna examples. Inset: Corresponding scattering cross-section spectra. Approaching emission strengths are observed between the two antennas around the telecom wavelengths.

The synergy of graphene and plasmonic antennas holds the promise to help us venture into new territories of tunable photonic signal manipulations at nanoscale. To achieve the design goal of wavelength-tunable, highly directional, polarization sensitive and mode selective on-chip optical coupling devices, a library of full-vector calculations are performed via Finite-Difference Time-Domain (FDTD) method. We firstly start with the analysis of single antenna elements loaded with graphene. Figure 1(a) illustrates the basic configuration, where a cuboid antenna is placed on an infinitely large layered substrate. The substrate is composed of a graphene monolayer, a 5 nm-thick hexagonal boron nitride (h-BN) and an immensely thick silicon substrate from top to bottom. We here fix the antenna height *h* as 50 nm and study how the choice of other geometry parameters influence scattering attributes. Figure 1(b) and (c) illustrate the normalized scattering cross-section *σ*_sca_ and phase response of the nanoantenna as a function of antenna length *l* respectively, under an exemplary instance of fixed antenna width as *w* = 50 nm. Figure 1(g) shows the corresponding resonant wavelengths to Fig. 1(b). Generally, a longer antenna length or larger aspect ratio will redshift the resonant wavelength^1^. Under constant excitation wavelength, the antenna impedance varies from capacitive to resistive and then to inductive across a resonance with the increment in antenna length, accompanied by a relative phase shift approximatly from 0 to *π*^5^.

Furthermore, we analyzed the tunability of graphene-loaded gold antenna elements. The solid lines in Fig. 1(d) show the variation of *σ*_sca_ under different *μ*_*c*_ of an antenna structure of *l* × *w* × *h* = 160 × 20 × 50 nm. The resonant wavelength *λ*_res_ decreases as *μ*_*c*_ increases. An approximate 90 nm shift in *λ*_res_ is observed if we tune *μ*_*c*_ from 0 to 2 eV. Moreover, by comparing the purple dashed line and the solid lines, one can notice that despite of its atomic thickness, the introduction of graphene also slightly promotes the resonance scattering intensity at higher chemical potential^20^. The green dotted line depicts the *σ*_sca_ spectrum of a broader antenna (*l* × *w* × *h* = 160 × 50 × 50 nm) and a stronger scattering intensity plus blueshifted resonant wavelength is exhibited. h-BN is selected as the spacer material for it can maintain the high carrier mobility of graphene and reduce the device’s total capacitance-resistance (RC) parameter^34,39^. Moreover, it provides electrical insulation and encapsulate graphene from environmental contaminations. As is illustrated in Fig. 1(e) and (f), the spacer layer also brings about a favorable near-field enhancement and increased scattering intensity^22–24^. The underlying tuning mechanism can be mainly attributed to the variation of graphene’s permittivity upon the electrical gating^20^ instead of the modulation of load between antenna ends^22,40^, while the influence of its tunable intrinsic surface plasmon polaritons will only become pronounced at lower frequencies. Meanwhile, compared with the proposal of utilizing nematic liquid crystals to tune the optical antennas^40^, graphene exhibits excellent comspatibility with nanofabrication and can operate in a more compact and swift manner.

To achieve directional coupling, a dipole model is applied for theoretical analysis^5,18^. Taking an antenna array comprised of two antenna elements as an instance, the antenna radiations can be approximated as the combination of two dipole sources *P*_*a*_ and *P*_*b*_ with phase delays of *α*_*a*_ and *α*_*b*_ accordingly: *P*_*a*_ = *A*exp[*i*(**k** ⋅ **r** + *α*_*a*_)], *P*_*b*_ = *B*exp[*i*(**k** ⋅ **r** + *α*_*b*_)]. Here *A* and *B* represent radiation amplitudes and are connected to the amplitude responses of antenna elements. As is illustrated in Fig. 1(h), a propagation phase stemmed from the relative displacement of the two dipoles is set as *α*_*p*_. If destructive interference is achieved in the right side as

1 $$({\alpha }_{a}+{\alpha }_{p})-{\alpha }_{b}=\pi $$

directional emission to the left takes place. Similarly, if the relative phase difference satisfies

2 $$({\alpha }_{b}+{\alpha }_{p})-{\alpha }_{a}=\pi $$

scattering with high directionality to the right side can be realized. We first fix the antenna width and height at 50 nm and vary the length of the antenna. Two gold antennas with lengths of 160 and 210 nm are demonstrated here as an example. As is shown in Fig. 1(i), their phase retardation difference at 1.55 μm is calculated as Δ*α* = *α*_*a*_ − *α*_*b*_ = −75°. As we will show later, the two antennas can be effectively leveraged as compact couplers by properly design their displacement and orientations. Generally, the wavelength of directional scattering locates between the resonant wavelengths of the two antennas.

Here we note that though the abovementioned design procedures are conducted for antennas located on an infinitely large substrate, they are applicable for antennas sitting on waveguides^7,8^. By extracting the field distributions between simulation pairs of a bare waveguide and the same waveguide patterned with antennas, one can get the exact phase and amplitude responses of the nanoantennas, which remain essentially identical as those obtained from antennas placed on the interface between two semi-infinite media^7^. The near-field couplings between adjacent antennas are not taken into account here in these simulations. The coupling effects in between adjacent antennas will generally redshift the operation wavelength but do not exert significant perturbations as numerical optimizations will be performed to find the device structure with optimal parameters.

### TE mode couplers with single group antennas

**Figure 2:**
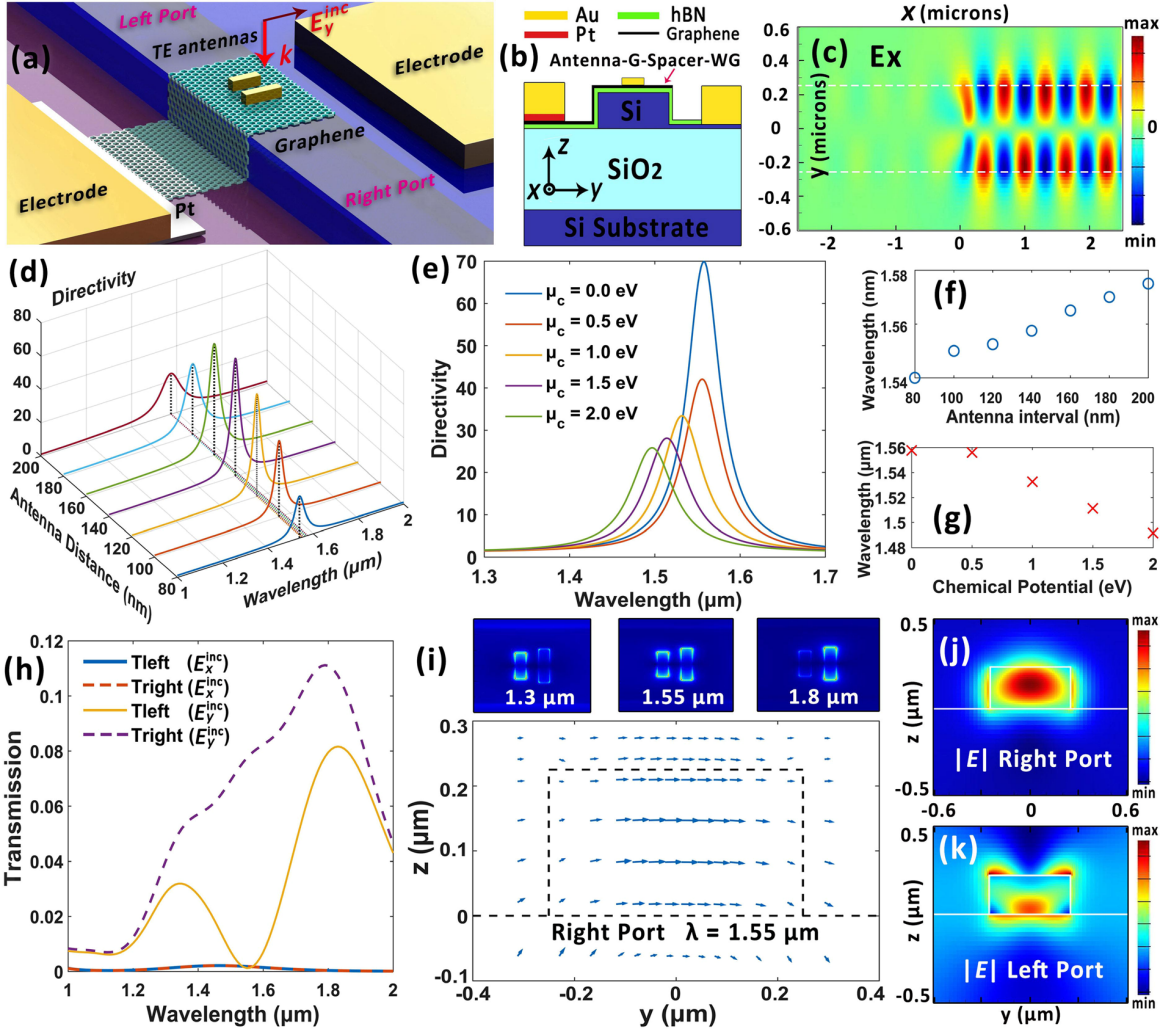
Single group TE antenna coupler. (**a**) Perspective view of the proposed device for directional coupling *y*-polarized incident light into fundamental TE mode to the right port around 1.55 μm. (**b**) Cross-sectional view of the graphene-hybrid waveguide patterned with gold nanoantennas. Inset: System coordinates. (**c**) Ex distribution along the waveguide central plane at 1.55 μm. (**d**) Directivity as a function of antenna center-to-center distance along *x* axis. (**e**) Tunability analysis of the proposed device. (**f**) and (**g**) Corresponding center operation wavelength *λ*_*d*_ to (**d**) and (**e**) respectively. (**h**) Transmission rate of the waveguide ports under different incident polarization states. (**i**) Vector-graph of the electric field distribution at the right port. Inset: $|{\bf{E}}|$ distribution details for the antennas along the graphene plane. (**j**) and (**k**) $|{\bf{E}}|$ distributions for the right and left port respectively.

Figure 2(a) illustrates the schematic view of the proposed device to achieve tunable, highly directional, mode selective and polarization selective couplings from linearly *y*-polarized incident light into right-propagating fundamental TE mode. The waveguide cross-section is 500 × 220 nm that roughly meets the fundamental mode transmission condition around telecommunication wavelengths. A 5 nm-thick h-BN spacer layer coats the waveguide and isolates graphene from silicon back gate^34^. The antennas sit directly on the graphene monolayer^21–23^ and the Pt/Au electrode is applied to reduce contact resistance^41^. Two exemplary gold antennas with identical width and height of 50 nm but different lengths of 160 and 210 nm are applied to constitute the antenna array (namely single group TE antennas). Figure 2(b) depicts the cross-sectional view of the hybrid waveguide with inset coordinates, where the antenna centers are aligned along *x* axis and the graphene-spacer-silicon capacitor configuration is applied to tune the chemical potential of graphene *μ*_*c*_.

According to the design analysis after Eq. 2, the phase difference between the two antenna elements is Δ*α* = −75° at *λ*_0_ = 1.55 μm. Taking the effective index of fundamental TE mode as *n*_eff_ = 2.46, to realize directional emission to the right port at *λ*_0_ = 1.55 μm, the optimal antenna center-to-center interval is predicted as *dx* = (*π* + Δ*α*)/*k* = 184 nm, where *k* = *n*_eff_*k*_0_ = 2*πn*_eff_/*λ*_0_ is the effective wavevector in silicon waveguide. It is close to the optimized value of 160 nm obtained by parametric sweep shown in Fig. 2(d). In Fig. 2(c) we plot the distribution of electric field component **E**_*x*_ in the proposed waveguide under normal irradiance of *y*-polarized light source, where unidirectional mode-selective coupling is achieved around 1.55 μm. If we define the directivity parameter as the ratio of optical power transmitted through the two ports in opposite directions (namely left port and right port), a directivity of 70 is attained at 1.55 μm under *μ*_*c*_ = 0 eV. Notably, this flexible coupling is attained within an extremely compact area of only 210 × 210 nm^2^.

The tunability analysis is illustrated as Fig. 2(e). Here we define the central wavelength of the directivity peak as the operation wavelength *λ*_d_ and its variations are shown in Fig. 2(g) accordingly. A spectral tunable range of 67 nm with an average bandwidth (FWHM) of 56 nm is achieved via tuning the chemical potential *μ*_*c*_ from 0 to 2 eV. Figure 2(h) depicts the normalized transmittances of the waveguide ports under different incident polarization states. A coupling efficiency of 8% (which remains an acceptable value considering the ultracompact volume $\sim {\lambda }^{3}\mathrm{/1600}$ of the antenna array) to the right-propagating TE mode is realized at 1.55 μm under *y*-polarized incident light (${{\bf{E}}}_{y}^{{\rm{inc}}}$). An extremely high polarization sensitivity is also exhibited, as the antennas can only be effectively excited when incident electric filed component is parallel to its long axis. Moreover, the transmission curves of the left (*T*_left_) and right port (*T*_right_) can be comprehended as the superposition of two antenna radiations. As is shown in Fig. 2(i) inset, the short antenna reaches its resonance around 1.3 μm and corresponds to the first radiation peak of ${T}_{{\rm{left}}}({{\bf{E}}}_{y}^{{\rm{inc}}})$ in Fig. 2(h). The longer antenna is responsible for the second transmission peak that gets pronounced at 1.8 μm. At operation wavelength of 1.55 μm, the two antennas are almost equally excited and their destructive interference results in the near-zero transmission rate at the left port. Figure 2(i) and (j) illustrate the vector diagram of the calculated electric field **E** and $|{\bf{E}}|$ distribution respectively for the right port at 1.55 μm. A good fundamental TE mode is oberved, validating the mode-selective coupling with fine quality. Figure 2(k) shows the $|{\bf{E}}|$ distribution at the left port featuring the asymmetric coupling characteristic, which can also be comprehended via the phase offset or the effective unidirectional momentum provided by the antenna array that leads to asymmetric phase matching conditions in opposite directions^8^.

### TM mode couplers with single group antennas

**Figure 3:**
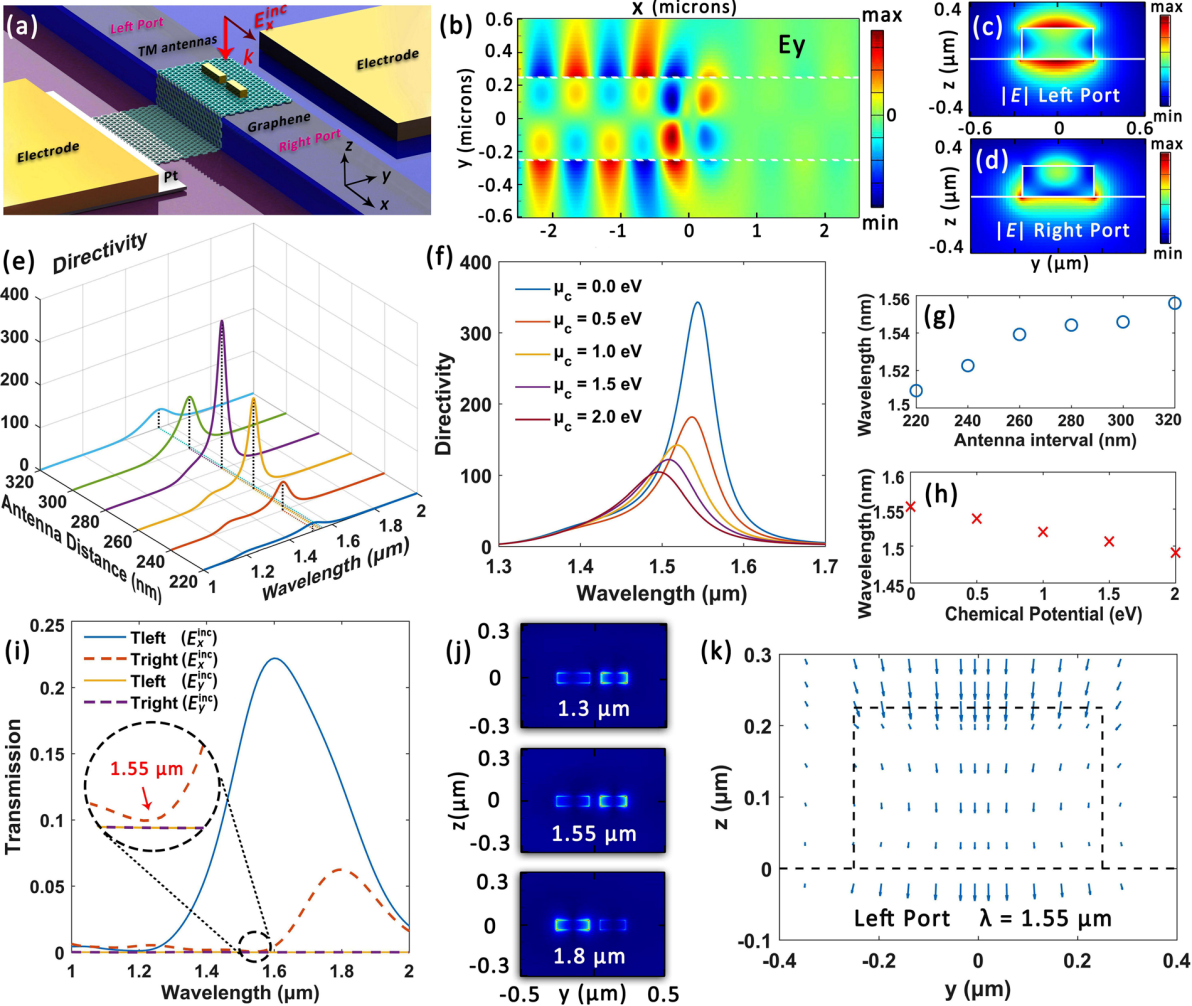
Single group TM antenna coupler. (**a**) Schematic of the device for coupling *x*-polarized incident light into left-propagating fundamental TM mode around 1.55 μm. (**b**) Distribution of electric field component **E**_*y*_ along the middle waveguide plane. (**c**) and (**d**) Electric field norm distribution for the left and right port respectively at 1.55 μm. (**e**) and (**f**) Directivity as a function of antenna center-to-center interval and graphene chemical potential respectively. (**g**) and (**h**) Corresponding operation wavelength variations for (**e**) and (**f**) respectively. (**i**) Normalized transmission curve for the two waveguide ports under different excitation polarization states. (**j**) $|{\bf{E}}|$ distribution details along graphene plane. (**k**) Corresponding vector map for (**c**).

Ultracompact on-chip coupling antennas that highly directionally couples incident light into fundamental TM mode can be implemented similarly. Figure 3(a) illustrates the schematic of the hybrid waveguide pattered with horizontally orientated gold antennas (namely single group TM antennas). The waveguide structure is the same as that in Fig. 2(b), whereas the incident source is changed into *x*-polarized light. The antenna width and height are also identically fixed as 50 nm in this example and the numerically optimized antenna lengths are 220 nm (left) and 170 nm (right) respectively. They locate at the waveguide center with their long axis aligned along *x* direction. The phase difference between the two antenna elements at 1.55 μm is Δ*α* = *α*_*a*_ − *α*_*b*_ = 78°. Taking the effective index of fundamental TM mode as ${n}_{{\rm{eff}}}^{\prime} =1.79$, the anticipated center-to-center distance of the two gold antennas is ${d}_{x}=(\pi -{\rm{\Delta }}\alpha )/({n}_{{\rm{eff}}}^{\prime} {k}_{0})=245$ nm, generally matching the numerically optimized value of 280 nm. Here the effective area for coupling is within 475 × 50 nm^2^, which is highly applausive for on-chip signal applications. Figure 3(b) depicts the distribution of electric field component **E**_*y*_ along the waveguide central plane and highly directional left-coupling is achieved. According to Fig. 3(c) and (k), the electric field distribution at the left port is in excellent agreement with a fundamental TM mode. In Fig. 3(e) and (f) we plot the directivity parameter as a function of antenna interval and the chemical potential of graphene *μ*_*c*_ respectively. The corresponding variations of operation wavelength *λ*_d_ are shown in Fig. 3(g) and (h) accordingly. By altering *μ*_*c*_ from 0 to 2 eV, the operation wavelength of the proposed device can be tuned across 64 nm and the maximum directivity reaches 346 (25 dB). Figure 3(i) depicts the transmission rate (normalized to source power) of the waveguide ports under different incident polarization states under *μ*_*c*_ = 0 eV. An extremely high polarization-sensitivity is also observed. The coupling efficiency at 1.55 μm is 18%, which remains a relatively big value for the array of only two subwavelength antennas. The electric filed norm distribution detail around the antennas in the graphene plane is shown in Fig. 3(j) accordingly, where relatively stronger couplings between the antenna elements are expected.

Here the orientation of the antenna long axis is parallel to the waveguide and light propagation direction, which leads to a coupling from *x*-polarized incident light into TM mode. This phenomenon can be comprehended via the spatial overlap between the antenna radiations and target waveguide mode^7^. For the formerly elaborated TE antennas that are perpendicular to the waveguide orientation, their dominant near-field electric component **E**_*y*_ exhibits good overlap with the fundamental TE mode. In contrast, for the TM antennas **E**_*y*_ is the least pronounced, while its electric field components possess better spatial overlap with the TM mode and therefore lead to effective couplings. Consequently, selective mode coupling or conversion can be implemented via engineering the antenna structure and orientations.

## Discussion

One may notice that the coupling efficiencies for the aforementioned proposals are not very high or desire the wavelength-tunable range to be further expanded. Here we elucidate that both of these parameters can be further ameliorated by introducing more antennas to the array or modifying the geometry deign of antenna elements. Two more supplementary design scenarios are presented as follows.

### TE mode couplers with double group antennas

**Figure 4:**
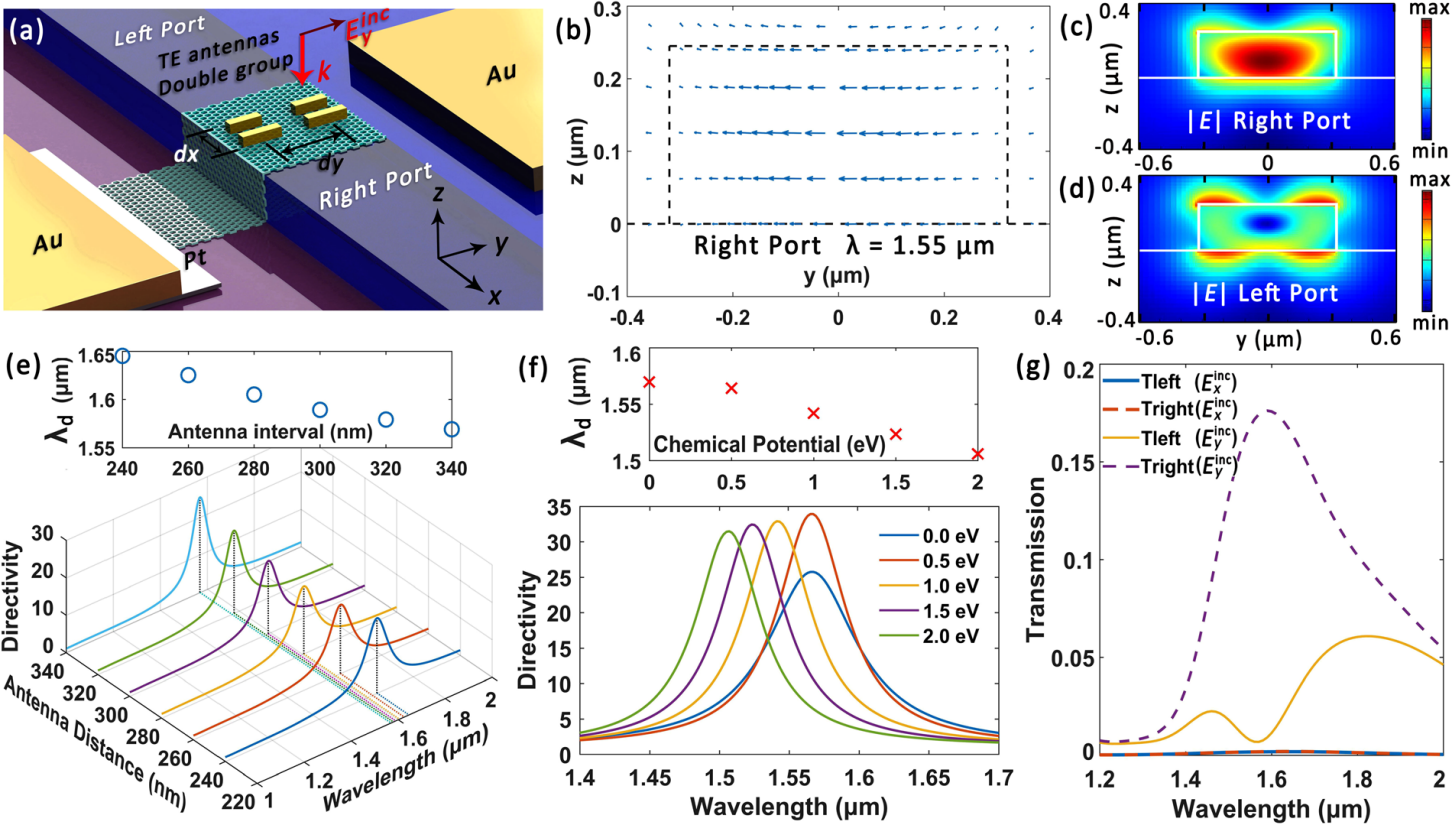
Double group TE antenna coupler. (**a**) Schematic of the device for right-propagating TE mode couplings. (**b**) Vector diagram for the electric field at the right port. (**c**) and (**d**) Electric field norm distribution for the right and left port at 1.55 μm respectively. (**e**) and (**f**) Directivity as function of antenna group distance *dy* and *μ*_*c*_ respectively. Insets: Variations for operation wavelength *λ*_d_. (**g**) Normalized transmission rate for the waveguide ports under different polarization states.

Figure 4(a) shows the structure sketch of the graphene-laminated silicon waveguide patterned with double group TE antennas, which couples *y*-polarized incident light into right-propagating TE mode with high directionality. Here we emphasis that we focused on the optimization of the directivity and tunability parameter and the simultaneous realization of an accredited mode quality in the output port, while increasing the coupling efficiency is not one of the targets. Though the overall efficiency of a single subwavelength antenna may remain compromised, it can be improved via adding more antennas to the array. The same waveguide configuration but with a larger cross-section of 640 × 240 nm is applied there to accommodate more antennas. The antenna array contains two identical groups of antennas and each group consists of two gold antennas of different lengths: 165 nm (left short antenna) and 200 nm (long antenna at the right side). The width and height of all the antennas are also fixed as 50 nm here. The center-to-center distance between the antennas in the *x* direction *dx* is 160 nm, while the group interval in *y* direction *dy* is 340 nm.

According to Fig. 4(b) and (c), an electric field distribution in good agreement with fundamental TE mode is observed in the right port. A hybrid mode distribution featuring asymmetric coupling is also recorded for comparison in Fig. 4(d). In Fig. 4(e) we plot the variation of the directivity parameter as a function of group interval *dy*. A bigger *dy* slightly decreases the operation wavelength *λ*_d_ as the antenna group interval influences the near-field coupling between antennas. Figure 4(f) illustrates the tunability analysis of the proposed device, where *λ*_d_ can be tuned across 63 nm by altering *μ*_*c*_ from 0 to 2 eV. Figure 4(g) shows the transmission rate (normalized to source power) under different incident polarization states. The coupling efficiency at 1.55 μm is ameliorated to about 18% while its high polarization sensitivity is still preserved.

### Couplers patterned with heterogeneous antenna array

In the above discussions, the width and height of the antennas are all fixed at 50 nm, leaving the length as only variable for antenna elements. Here we demonstrate that the tunable range of the proposed couplers can be further expanded to largely transcend the operation bandwidth, by flexibly applying antenna pairs with different lateral dimensions (namely heterogeneous antennas).

**Figure 5:**
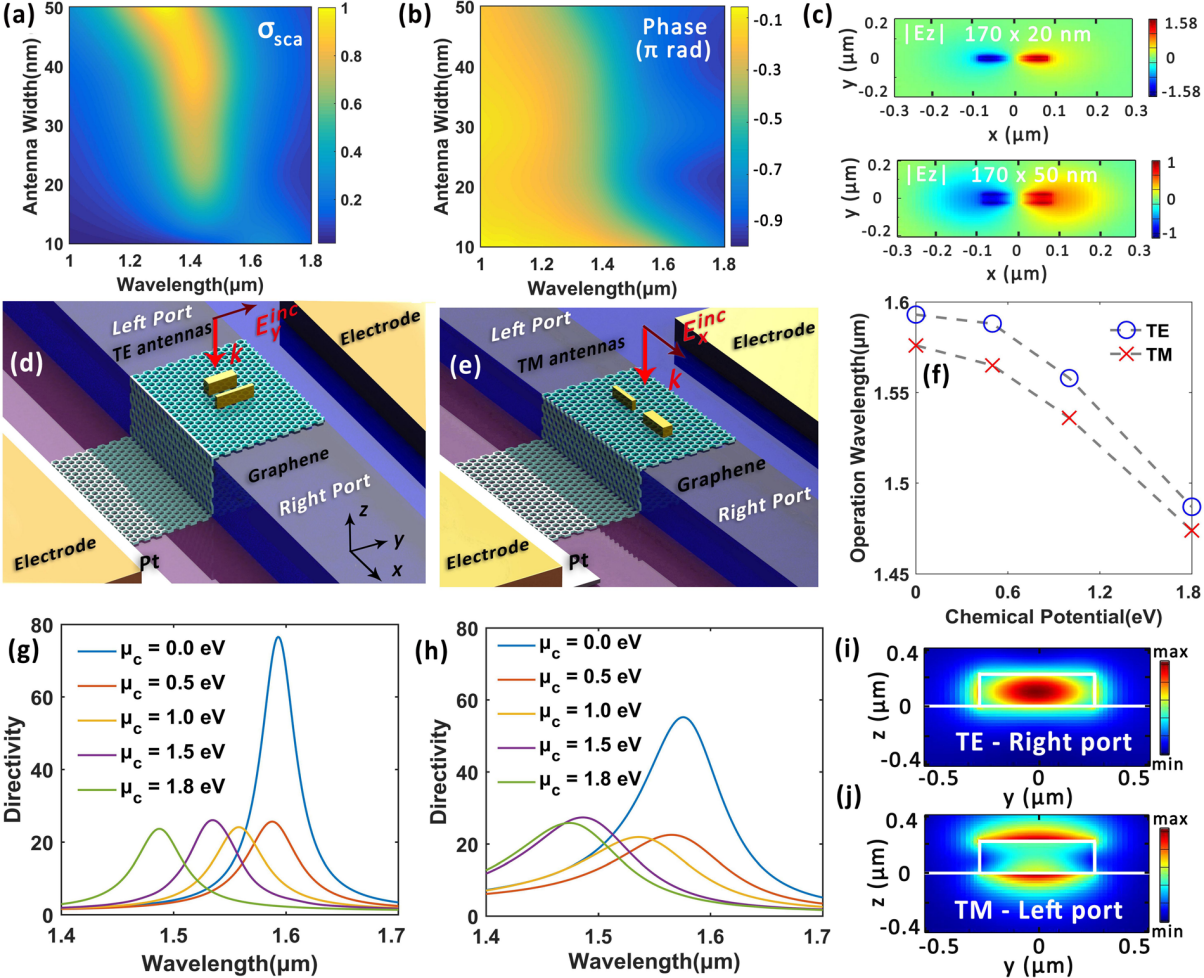
(**a**) and (**b**) Normalized scattering cross-section and phase response as a function of antenna width (fixed *l* = 170 nm *h* = 50 nm and spacer layer thickness 10 nm) under *μ*_*c*_ = 0.2 eV. (**c**) Comparison of the $|{{\bf{E}}}_{z}|$ distribution for two gold antennas with different aspect ratio. (*l* × *h* × *w* = 170 × 50 × 20 nm and *l* × *h* × *w* = 170 × 50 × 50 nm for the upper and lower panel respectively). (**d**) and (**e**) Device structures for directional couplings to left-propagating TE and right-propagating TM mode respectively. The antenna center-to-center distances are 100 nm (**d**) and 240 nm (**e**). (**f**) Tunability illustration of central operation wavelength *λ*_d_ corresponding to (**d**) and (**e**). (**g**) and (**h**) Directivity curves under different *μ*_*c*_ for devices sketched in (**d**) and (**e**) respectively. (**i**) and (**j**) $|{\bf{E}}|$ distributions of at the left port for (**d**) and right port for (**e**) respectively at 1.55 μm.

We pick two sets of data from the library generated from the aforementioned parametric sweep elaborated for Fig. 1(a) where the thickness of h-BN spacer is increased to 10 nm. Figure 5(a) and (b) illustrate the scattering cross-section and phase response of the antenna element respectively. The antenna length and height are here fixed as 170 nm and 50 nm respectively but the antenna width is varied from 10 to 50 nm. The plasmonic resonance in a slender antenna can be decomposed into two modes: the longitudinal mode parallel to the long axis and the transverse mode perpendicular to the long axis. A red-shift in resonant wavelength is observed when decreasing antenna width, for the transverse modes are suppressed in thinner antennas with higher aspect ratio^42^. The goal of enlarged tunable wavelength range can be addressed by improving the sensitivity of antenna phase response to graphene’s chemical potential *μ*_*c*_. The scattering properties of the antennas are affected by the dielectric constant of the surrounding medium. As graphene is the only active material, the key lies in enhancing the electric field overlap between antenna and graphene to elevate their interactions. A stronger field enhancement is observed for the thinner antenna with sharp end for it can sustain more charges at antenna ends [shown as Fig. 5(c)], leading to enhanced sensitivity to the change in graphene permittivity (see Fig. S7(c) and (d) in the Supplementary Material for details).

An ameliorated tunable performance is achieved by combining a thin antenna (*l* × *w* × *h* = 210 × 20 × 50 nm) with a broad antenna (*l* × *w* × *h* = 170 × 50 × 50 nm) on top of the graphene hybrid waveguide. The schematic for the modified TE antennas that directionally couples *y* polarized incident light into right-propagating fundamental TE mode is shown in Fig. 5(d). Figure 5(e) sketches the structure for coupling *x* polarized incident light into left-propagating TM mode. The tunability performance of the operation wavelength *λ*_d_ is manifested in Fig. 5(f). According to Fig. 5(g) and (h), the tunable range of *λ*_d_ for the abovementioned couplers are 107 nm and 103 nm respectively. As is illustrated in Fig. 5(i) and (j), fine mode qualities with respective to TE and TM modes are observed at the right and left port for the TE and TM couplers respectively. Furthermore, if antennas with larger aspect ratio are applied, the device performance regarding directivity and wavelength tunability can be further ameliorated. Extended discussions on device design and potential applications are made in the Supplementary Material.

### Ultracompact tunable waveguide signal detectors with polarization-sensitivity

**Figure 6:**
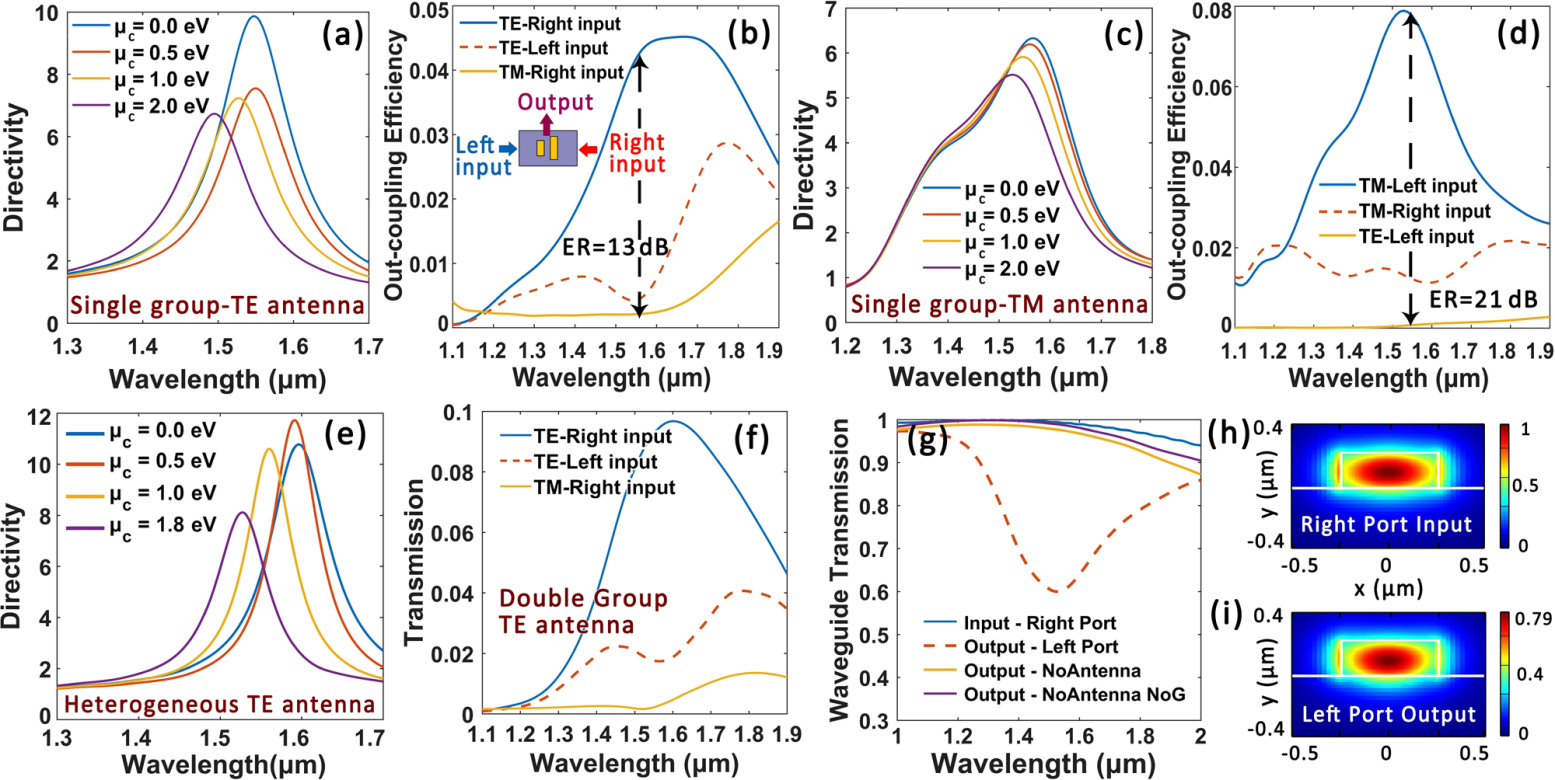
Ultracompact mode-selective directional waveguide signal detectors. (**a**) and (**c**) Tunability analysis for the proposed detectors under the same configurations of Figs 2(a) and 3(a) respectively. (**b**) and (**d**) Mode sensitivity analysis for (**a**) and (**c**) respectively. (**e**) Tunability analysis for the device sketched in Fig. S7(f). (**f**–**i**) Signal probing performance when fixing *μ*_*c*_ at 0 eV for the device sketched in Fig. 4(a). (**f**) Out-coupling efficiency spectrum. (**g**) Transmission rate of the bus waveguide. (**h**) and (**i**) $|{\bf{E}}|$ distribution at 1.55 μm for the right and left ports respectively.

The reciprocity nature of the proposed devices indicates that they can also be leveraged as compact waveguide signal detectors, which selectively couples only one polarization mode with specific propagation direction out of the waveguide and leaving other signal channels undisturbed. Here we input either fundamental TE or TM mode into the left or right port of the waveguide and monitor the scattered light power on the top of the antenna array. The schematic is sketched in Fig. 6(b) inset. Figure 6(a) and (b) illustrates the tunability analysis and output transmission rate of the proposed detector respectively, which is the same structure as that in Fig. 2(a). The central wavelength of the detected signal can be tuned across 59 nm with the corresponding out-coupling efficiency of 4.5%. As is shown in Fig. 6(b), only the injected fundamental TE mode can be effectively coupled out of the waveguide around telecommunication wavelengths. If we define the ratio of out-coupling efficiency between two orthogonal input fundamental modes as the extinction ratio (ER), an ER of 13 dB is achieved at 1.55 μm. Moreover, the out-coupling also exhibits relatively high directionality, as only right-to-left propagating TE mode is efficiently probed out of the waveguide at operation wavelength *λ*_d_ = 1.55 μm, while the coupling efficiency of the left-to-right propagating is suppressed at *λ*_d_.

The detection performances for TM antennas are shown in Fig. 6(c) and (d). The directivity parameter is relatively compromised but the out-coupling efficiency and ER are comparatively higher: 8% and 21 dB respectively at 1.55 μm. The tunable range of the central detection wavelength is 53 nm. Figure 6(e) illustrates the detection performance for the heterogeneous TE antennas sketched in Fig. S7(f) in the Supplementary Material, where λd can be tuned across 76 nm and an ER about 14 dB is attained. As is shown in Fig. 6(f), the out-coupling efficiency are improved to 11% for the configuration of double group TE antennas depicted in Fig. 4(a). Figure 6(g) shows the corresponding transmission rates of the hybrid waveguide to Fig. 6(f). The calculated insertion loss at 1.55 μm for *μ*_*c*_ = 0 eV is about 2 dB. Two simulations are performed for comparison, when the antennas are absent (but graphene is present, yellow solid line) and both the antennas and graphene are removed (purple solid line). Thanks to the ultracompact footprint of the antenna array (210 × 540 nm^2^), a small size of graphene flake can sufficiently modulate the antennas. Therefore, though graphene remains absorptive at *μ*_*c*_ = 0 eV, graphene-induced loss is still restricted at a low level (estimated below 0.4 dB)^31^. By comparing Fig. 6(h) and (i), one can notice that the proposed waveguide signal detector almost exert no influence on the mode quality of the probed optical signal. This mode-selective, propagation direction-sensitive, tunable compact waveguide signal detector can be utilized to selectively probe only one optical signal with specific polarization mode and propagation direction, without disturbing the transmission quality of adjacent signal channel states. Moreover, the central detection wavelength *λ*_d_ can be tuned by the bias voltages in an ultrafast manner, beneficial to further potential applications such as real-time on-chip non-destructive detection and intercept signal probing.

## Conclusions

Highly directional tunable on-chip waveguide couplers with mode-selectivity and polarization-sensitivity can be achieved with graphene-laminated photonic waveguides patterned with nanoatenna arrays. Versatile couplings are achieved in an ultracompact and flexible fashion by marrying the advantages of tight field confinement and the largely tunable carrier density of graphene. The incident linearly polarized light signals can be selectively coupled into either left- or right-propagating fundamental modes with good mode quality and high directionality up to 346 (25 dB). Notably, the couplings can be realized with a subwavelength area of only 210 × 210 nm^2^. Moreover, the coupling efficiency and tunable wavelength range can be further extended to 18% and 107 nm respectively around the telecom wavelength by employing more antennas and antenna pairs with different lateral geometry.

Furthermore, the proposed schemes can also be applied to ultracompact on-chip waveguide signal detectors, where the detection wavelength can be controlled flexibly with high speed in real time by the applied voltages. The detectors will only selectively extract a certain signal with specific mode and propagation direction while keeping the other optical channels undisturbed. Our designs can be further extended to hybrid dielectric antennas to mitigate insertion loss^43,44^ or exploring the epsilon near zero effect to strengthen tunability^45,46^, providing a positive paradigm upon tunable chip scale photonic applications, such as flexible couplings, light routing, multiplexing and sensing.

## Methods

The complex surface conductivity of graphene can be derived from Kubo Formula consisting of intra- and inter-band contributions^28,32–34^.

3 $$\begin{array}{rcl}{\sigma }_{s}({\mu }_{c}) & = & {\sigma }_{0}\frac{4{\mu }_{c}}{\pi \hslash ({\rm{\Gamma }}-i\omega )}+{\sigma }_{0}\{1+\frac{1}{\pi }\arctan (\frac{\hslash \omega -2{\mu }_{c}}{\hslash {\rm{\Gamma }}})\\  &  & -\frac{1}{\pi }\arctan (\frac{\hslash \omega +2{\mu }_{c}}{\hslash {\rm{\Gamma }}})-\frac{i}{2\pi }\,\mathrm{ln}[\frac{{(\hslash \omega +2{\mu }_{c})}^{2}+{(\hslash {\rm{\Gamma }})}^{2}}{{(\hslash \omega -2{\mu }_{c})}^{2}+{(\hslash {\rm{\Gamma }})}^{2}}]\}\end{array}$$

Here *σ*_0_ = *e*^2^/(4*ℏ*) refers to the optical conductivity of undoped graphene; *ω* is angular frequency and *ℏ*Γ = 5 meV^34^ stands for the charged particle scattering rate. As graphene supports largely tunable carrier densities *n* around^47,48^ 10^15^–10^18^ m^−2^, the Fermi level can be equivalently tuned following the equation ${E}_{F}=\hslash {v}_{F}\sqrt{\pi n}$^48^. Taking the Fermi velocity of graphene on h-BN buffer layer as *v*_*F*_ = 1.5 × 10^6^ m/s^39^, the Fermi level or chemical potential is estimated to be modulated to approximate 1.8 eV^49,50^. For telecommunication wavelengths around 1.55 μm, interband absorption of graphene is dominating. When the chemical potential *μ*_*c*_ is tuned to block interband transitions as a consequence of Pauli blocking^28^, the optical absorptions of graphene will drop drastically^31–33^ and graphene’s equivalent permittivity will undergo dramatic variations.

A set of full-vector FDTD simulations is performed to analyze the device performance. The abovementioned systems are embedded in coating material such as polymethyl methacrylate (PMMA)^51^ with a refractive index around 1.48. For the simulations regarding single antenna elements, a plane wave (as a representative example) is launched from the top and irradiates the gold antenna in normal direction. The incident linearly polarized electric filed component is aligned with the longer axis of the nanoantenna. The phase responses of the antennas are retrieved via monitoring the transmitted light in the silicon substrate after phase compensation^7,8^. Perfectly Matched Layers (PML) was applied to all the simulation boundaries. Graphene is taken into consideration by applying its complex surface conductivity to the boundary conditions^24^. For the simulations regarding the compact couplers and waveguide signal detectors, the total-field scattered-field (TFSF) light source^52^ is applied (see the Supplementary Matrial for details). The coupling efficiency is defined as the ratio of the total transmitted power through one certain waveguide port to the total power of the light source. The coupling efficiencies under the illumination of focused Gaussian beams with different values of objective nuerical aperture and the extended discussions are also presented in the Supplementary Material. Further calculations show that the wavelength-tunable range is almost identical between different light sources and the high directivity of our proposed devices is also maintained under the excitation of Gaussian beams. The antennas are located on the top of monolayer graphene (1.5 μm-long rectangular sheet with identical width as the waveguide). The left and right waveguide ports are located 5 μm away from the center of antenna arrays. A thickness of 5 nm is selected for h-BN spacer layer for it mitigates the driving voltage to tune graphene’s carrier density^31,34^. The Pt/Au electrical contact is adopted for its low contact resistance with graphene^32^. Thanks to the ultrahigh carrier mobility of graphene, the proposed devices are expected to possess ultrafast operation speed^28^.

## Electronic supplementary material

Supplementary information

## Data Availability

Data for the analysis are all included in this article and are available from the corresponding author upon reasonable request.
