# Supplementary material for: Ultracompact Graphene-Assisted Tunable Waveguide Couplers with High Directivity and Mode Selectivity
Source: Sci Rep. 2018 Sep 6;8:13362. doi: 10.1038/s41598-018-31555-7 (PMC6127104; doi:10.1038/s41598-018-31555-7)
Supplement: Supplementary file 1 — Supplementary information [file 41598_2018_31555_MOESM1_ESM.pdf]

Ultracompact Graphene-Assisted Tunable Waveguide Couplers with High Directivity and Mode Selectivity: Supplementary Information

Yuan Meng[1], Futai Hu[1], Yijie Shen[1], Yuanmu Yang[1], Qirong Xiao[1], Xing Fu[1], and Mali Gong[1,2,*]

[1]State Key Laboratory of Precision Measurement Technology and Instruments, Department of Precision Instrument,Tsinghua University, Beijing 100084, China
[2]State Key Laboratory of Tribology, Department of Mechanical Engineering, Tsinghua University, Beijing 100084, China
[*]gongml@mail.tsinghua.edu.cn

ABSTRACT

This document provides supplementary information to “Ultracompact Graphene-Assisted Tunable Waveguide Couplers with High Directivity and Mode Selectivity”. It includes the numerical calculation details and extended discussions on device designs and applications.

Numerical simulation details

Full vector finite-difference time-domain (FDTD) simulations are performed to calculate the electromagnetic fields. The Au (gold) material is modeled by the literature values of refractive index data considering dispersion by Johnson and Christy[1]. The refractive indices of silicon (Si) and silica substrate (SiO_2) are taken from Palik[2]. The refractive index of h-BN spacer layer is retrieved from the experimentally measured value[3] around the telecommunication wavelength.

The transmission rates of the waveguides are defined as the ratio of the total transmitted power through the waveguide port to the total power of the incident light source. Plane monitors (perpendicular to x axis with their sizes larger than the waveguide cross-section to fully accommodate the evanescent tails outside the waveguide) are applied to integrate the total Poynting vector to calculate the total transmitted power through the Left or Right Port of the waveguide. The coupling efficiency is then determined by the power transmission rate of the certain waveguide port for directional coupling.

The linearly-polarized total-field scattered-field (TFSF) light source[4–6]with plane wavefront is applied in the numerical simulations. Inside the TFSF boundary, the electromagnetic field is the sum of incident light source and scattered fields, where the antennas are equivalently excited by a plane wave. While outside the TFSF boundary, the influence of the light wave from the source will be excluded and only the scattered lights are analyzed. This source offers a convenient and accurate tool for the characterization and analysis of the antennas and the mode quality at waveguide ports. TFSF sources are commonly utilized to analyze the scattering properties of the antennas[6] and can be also applied in the calculations regarding the coupling efficiency of waveguides[7] and dispersive, lossy layered structures[8]. In our simulations, the TFSF boundary is extended from top coating medium (PMMA) to the silicon waveguide layer and to the bottom silica substrate without touching the waveguide boundaries or the Perfectly Matched Layers (PMLs). The device configurations and the implementation of graphene (two-dimensional sheet in surface conductivity model) are elaborated in the Method Section of the main text.

Device performance under the illumination of focused Gaussian beams

A set of supplementary calculations are performed for comparison, where the TFSF source is replaced by focused Gaussian beams. As tightly focused beams are considered here, full vectorial Gaussian beams are applied, for the scalar Gaussian beam approximation of the electric field remains valid only when the waist beam diameter is much larger than the diffraction limit. Figs. S1-S3 show the calculated results of coupling efficiency under the illumination of focused Gaussian beams with different values of the objective’s numerical aperture (NA) for the design examples of TE and TM mode couplers with single group antennas and TE couplers with double group antennas respectively. The illumination case of a diffracting plane wave source (i.e. plane wave sheared by a rectangular aperture with the size of 1 μm × 1 μm and locates 1.2 μm above the antenna array) is also calculated for comparison.

Taking TE mode coupler with single group antennas (structure shown in Fig.2a of the main text) as an instance, the transmission spectrum of the waveguide ports under different kinds of light sources are similar with near-zero transmission of the Left Port achieved at 1.55 μm. For Gaussian beam illuminations, the coupling efficiency is dependent on the value of NA of the objective by which the Gaussian beams are focused. The reason is that peripheral incident electromagnetic waves that do not 'touch' the antennas actually barely make contributions to the coupled power through the waveguide port and drags down the coupling efficiency (for the coupling efficiency is normalized to the total source power). When the incident light beam is tightly focused, the total excitation power will be utilized in a more efficient manner.

The coupling efficiency under the illumination of TFSF source, focused Gaussian source with NA equals to 1.4 (Olympus 100× MPLAPON-Oil, index matching liquid can be applied considering that our devices are designed to be embedded in PMMA with a refractive index around 1.48), 0.95 (Olympus 100× M Plan IR objective), 0.7 and 0.5 are 8.2%, 7.8%, 4.7%, 3.1% and 1.2% respectively. Considering the deep-subwavelength area (210×210 nm^2) or the ultracompact volume ($\sim\lambda^3/1600$) of the antenna array, the coupling efficiency may remain an acceptable value. The coupling efficiency of the TFSF source is the highest for in this case the antennas are well excited within the TFSF boundary and the coupling efficiency is normalized to the lateral source area (about 1.2 μm × 0.45 μm), which is smaller than the Gaussian beam sport. Furthermore, as is elaborated in the main text, the coupling efficiency can be further enhanced by introducing more antennas to the array. For the instance of TE mode coupler with single group TE antennas, the coupling efficiency under the illumination of focused Gaussian beams with objective NA of 1.4, 0.95 and 0.7 are 13.7%, 9.2% and 5.9% respectively. The high directivity (exceeding 10 dB) and excellent mode quality (shown as Fig. S4) are also maintained.

Furthermore, the directivity and tunability of our proposed devises are also investigated under the incidence of focused Gaussian beams. Fig. S5 illustrates the directivity curves for the first three design scenarios in the paper main text. It can be seen that though the exact value of the directivity depends on the condition of the light source, the wavelengths that correspond to the maximum directivity are almost identical for different light sources for all the three examples. Fig. S6 shows the tunability analysis. The wavelength tunable ranges under different light sources are also highly approaching: 66 nm for TFSF source, 64 nm for Gaussian beam with NA = 1.4 [shown as Fig. S6(a)] and 63 nm for Gaussian beam with NA = 0.7 [shown as Fig. S6(b)].

Extended discussions on design scenarios and potential applications

In this section, one more design example and some potential applications are discussed. Firstly, if slender antennas with larger aspect ratios can be applied, the wavelength tunable range and directivity performance can be further enlarged. Fig. S7 shows the design examples of integrating two nanoantennas on a 5 nm h-BN spacer with a fixed length 160 nm and different widths as 10 nm and 50 nm. Similar to Fig.4 (a) in the main text, the normalized scattering cross-section and corresponding phase response are illustrated in Fig. S7 (a), (b). Compared to board antenna [Fig. S7(c)], the electric field amplitude is magnified at the interface of the 10 nm-wide gold antenna and graphene [Fig. S7(d)]. A stronger field enhancement is observed for the thinner antenna with sharp end for it can sustain more charges at antenna ends, leading to enhanced sensitivity to the change in graphene permittivity. Enhanced field confinement results in a pronounced field overlap between field scattered by antenna element and graphene layer, which leads to a helpful improved sensitivity to the chemical potential of graphene μ_c. As is shown in Fig. S7(e), the resonant wavelength roughly decreases when upgrading μ_c from 0 to 1.8 eV. The resonant wavelength of the thin antenna can be tuned about 110 nm, which is larger than tuning range of another broad antenna.

Improved sensitivity provides the toolbox for directional couplers with better performance. The schematic of proposed coupler structures is illustrated in Fig. S7 (f) and (g) for coupling y-polarization to TE mode and x-polarization to TM mode respectively. As mentioned above in main text, the lateral geometry change will bring about a consequent variation in tuning capacity and directivity performance. By applying such design structures, the tunable range of TE mode coupler and TM mode coupler can be increased to 105 nm and 115 nm respectively, which are 2.8 times and 4.2 times of their bandwidths (FWHM of the directivity spectrum) accordingly. The maximum directivity exceeds 840 (29 dB) in Fig. S7(j). As is illustrated in Fig. S7(k) and (l), fine mode qualities with respective to TE and TM modes are observed at the left ports for the TE and TM couplers respectively.

Secondly, our proposal can be leveraged as polarizers with tunable operation wavelength. If the incident light contains two orthogonal polarization components ($\mathbf{E}_x$ and $\mathbf{E}_y$ for instance), then only one polarization component will be directionally coupled to one certain directiony, considering the high polarization sensitivity of our proposed devices.

Furthermore, if we combines antenna groups with different orientation, the functionality of tunable polarization sorters or routers[9, 10] can be achieved. Fig. S8(a) depicts one of the possible schematics, where two pairs of identical antennas as shown in S7(f) and (g) with antenna spacings as $d_1 = 200$ nm, $d_2 = 280$ nm and a group interval Δ of 150 nm. Assuming the incident light contains two signal channels encoded on orthogonal polarization states, this device example can unidirectionally couple the $\mathbf{E}_y$ polarization component into left-propagating TE mode as shown in Fig. S8(b) and (f). Meanwhile, the orthogonal polarization component ($\mathbf{E}_x$) is directionally coupled to the right port of the waveguide into fundamental TM mode with good quality as shown in Fig. S8(c) and (f).

References

1. Johnson, P. B. & Christy, R. W. Optical constants of the noble metals. *Phys. Rev. B* **6**, 4370–4379 (1972).
2. Palik, E. D. *Handbook of Optical Constants of Solids.* (Academic press, 1998).
3. Bao, Q. & Loh, K. P. Graphene photonics, plasmonics, and broadband optoelectronic devices. *ACS Nano* **5**, 83–90 (2011).
4. Schneider, J. B. Plane waves in FDTD simulations and a nearly perfect total-field/scattered-field boundary. *IEEE Transactions on Antennas Propag.* **52**, 3280–3287 (2004).
5. Abdijalilov, K. & Schneider, J. B. Analytic field propagation TFSF boundary for FDTD problems involving planar interfaces: lossy material and evanescent fields. *IEEE Antennas Wirel. Propag. Lett.* **5**, 454–458 (2006).
6. Schneider, J. B. & Abdijalilov, K. Analytic field propagation TFSF boundary for FDTD problems involving planar interfaces: PECs, TE, and TM. *IEEE Transactions on Antennas Propag.* **54**, 2531– 2542 (2006).
7. Guo, Y. *et al.* Chip-integrated geometric metasurface as a novel platform for directional coupling and polarization sorting by spin-orbit interaction. *IEEE J. Sel. Top. Quantum Electron.* **24**, 4700107 (2018).
8. Olkkonen, J. FDTD scattered field formulation for scatterers in stratified dispersive media. *Opt. Express* **18**, 4380–4389 (2010).
9. Tanaka, Y. Y. & Shimura, T. Tridirectional polarization routing of light by a single triangular plasmonic nanoparticle. *Nano Lett.* **17**, 3165–3170 (2017).
10. Guo, R. *et al.* High-bit rate ultra-compact light routing with mode-selective on-chip nanoantennas. *Sci. Adv.* **3**, e1700007 (2017).

Additional information

Competing interests: The authors declare that they have no competing interests.

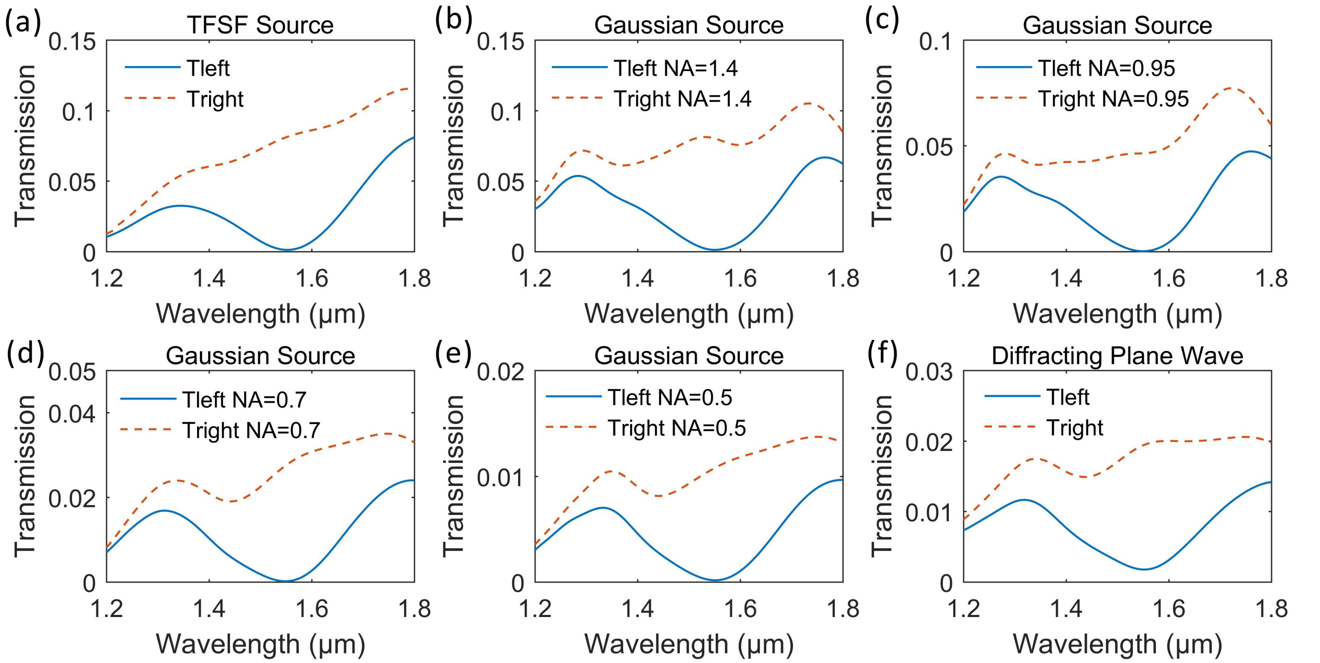

Fig. S 1. Waveguide transmission rates under different light sources for the TE mode couplers with single group antennas (corresponding to Fig.2a in the main text). (a) Total-field scattered-field (TFSF) source. (b)-(e) Focused Gaussian beams with the numerical aperture (NA) of the objective as 1.4, 0.95, 0.7 and 0.5 respectively. (f) The illumination under diffracting plane wave (PW, aperture size: 1 μm × 1 μm).

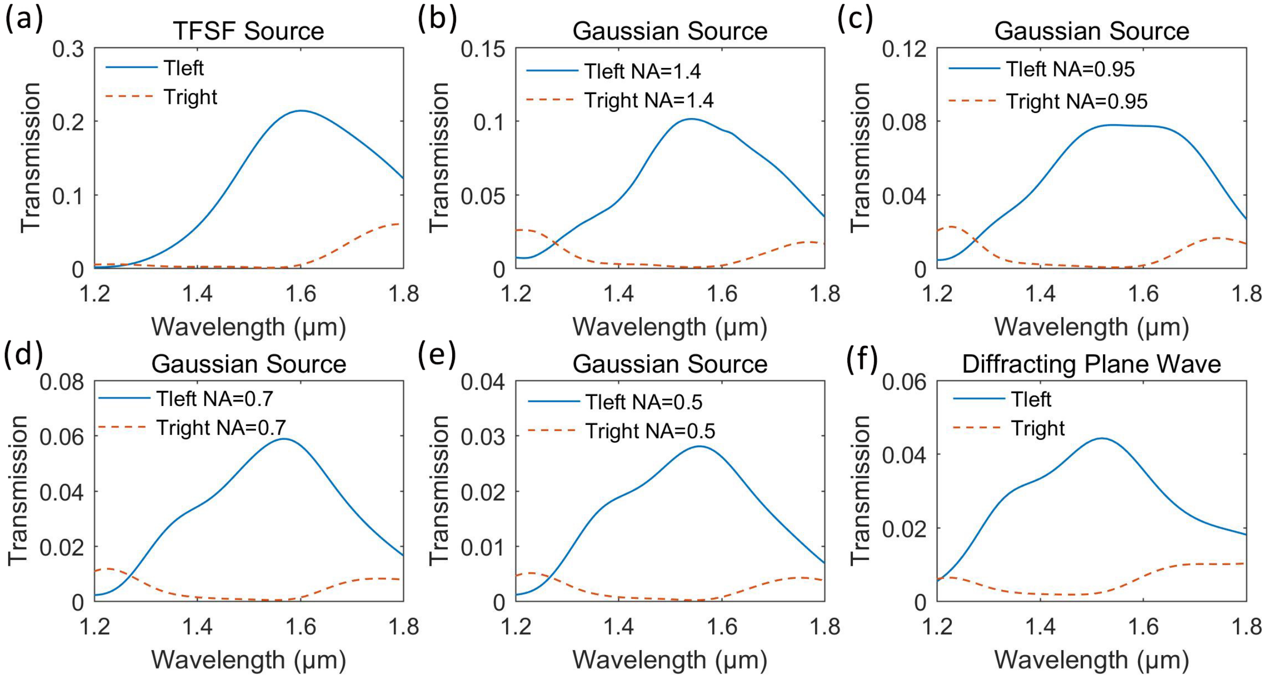

Fig. S 2. Waveguide transmission rates under different light sources for the TM mode couplers with single group antennas (corresponding to Fig.3a in the main text). (a) TFSF source. (b)-(e) Focused Gaussian beams with objective NA as 1.4, 0.95, 0.7 and 0.5 respectively. (f) Diffracting plane wave.

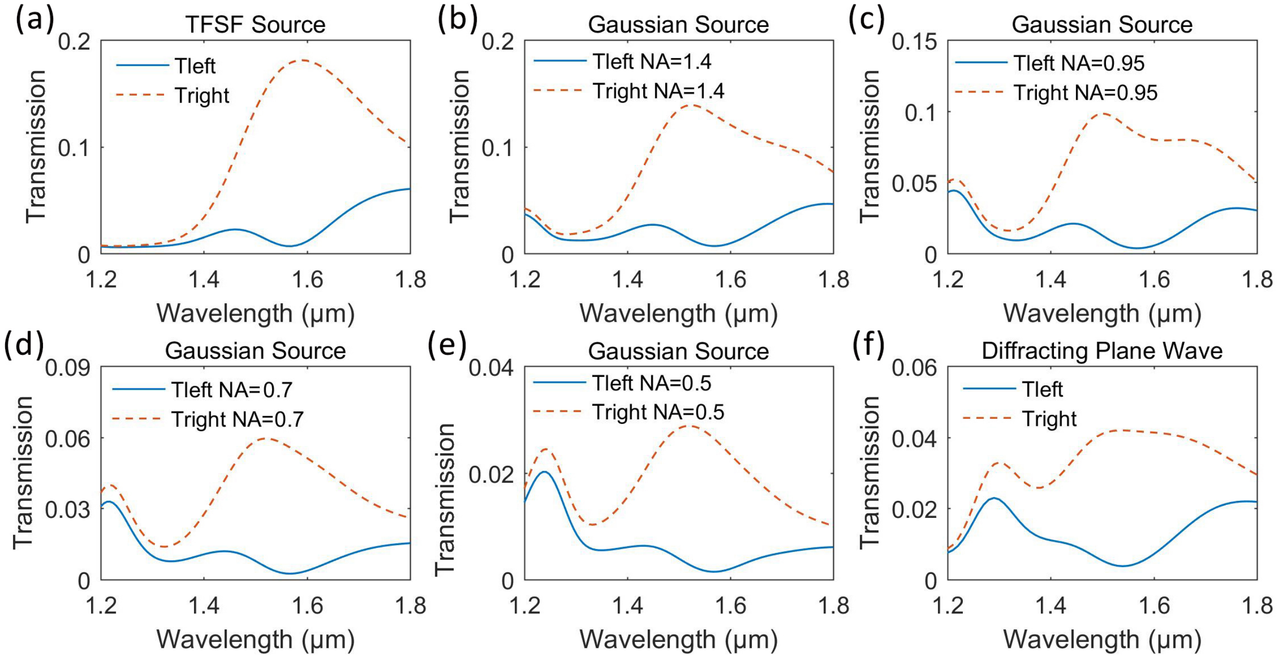

Fig. S 3. Waveguide transmission rates under different light sources for the TE mode couplers with double group antennas (corresponding to Fig.4a in the main text). (a) TFSF source. (b)-(e) Focused Gaussian beams with objective NA as 1.4, 0.95, 0.7 and 0.5 respectively. (f) Diffracting plane wave.

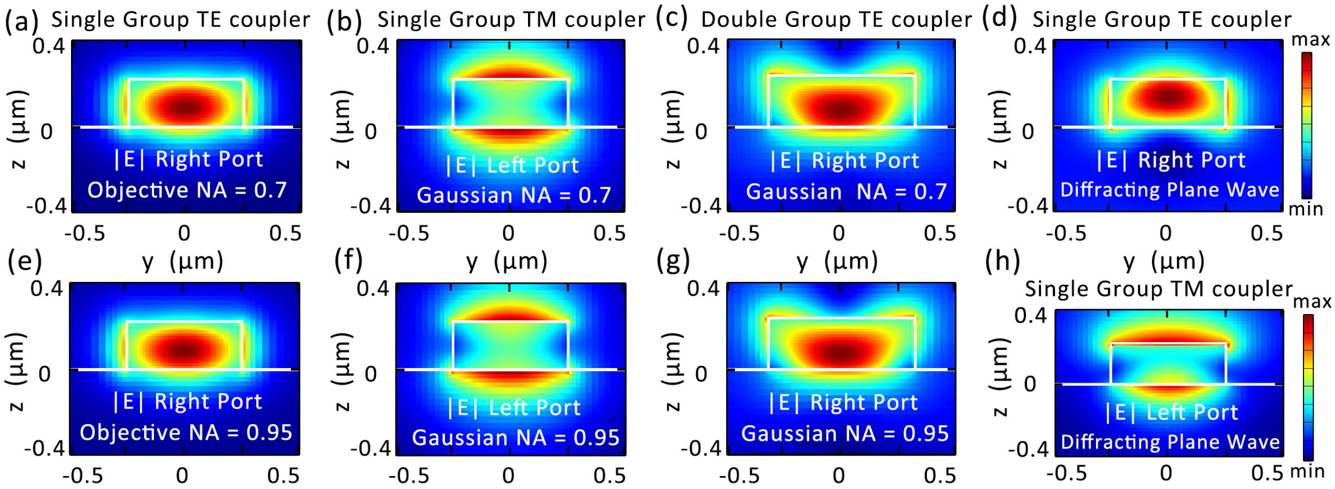

Fig. S 4. Electric field distributions for the waveguide ports for directional couplings under different illumination light sources under $\mu_c = 0$ eV.

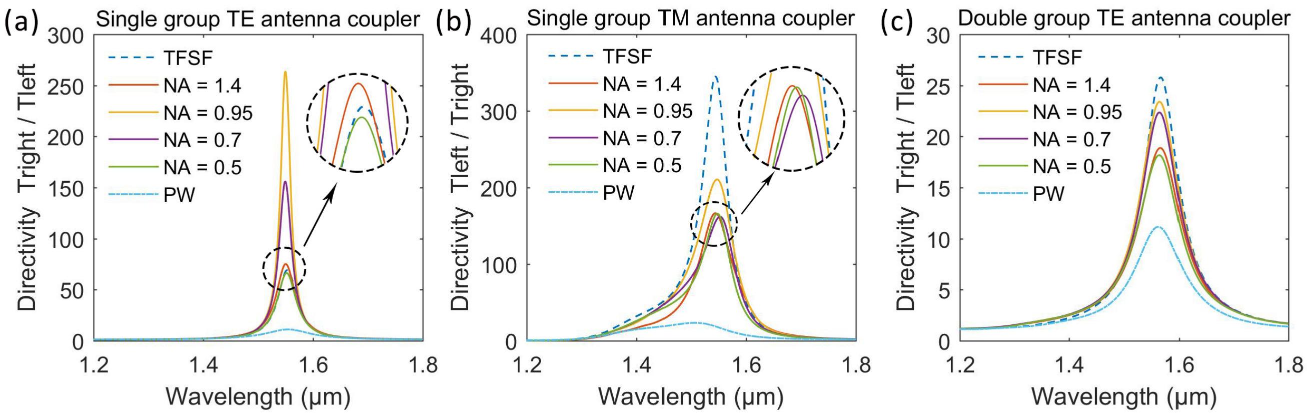

Fig. S 5. Comparison of the directivity parameter under different excitation sources for the design examples of TE (a) and TM (b) mode couplers with single group antennas and (c) TE mode coupler with double group antennas.

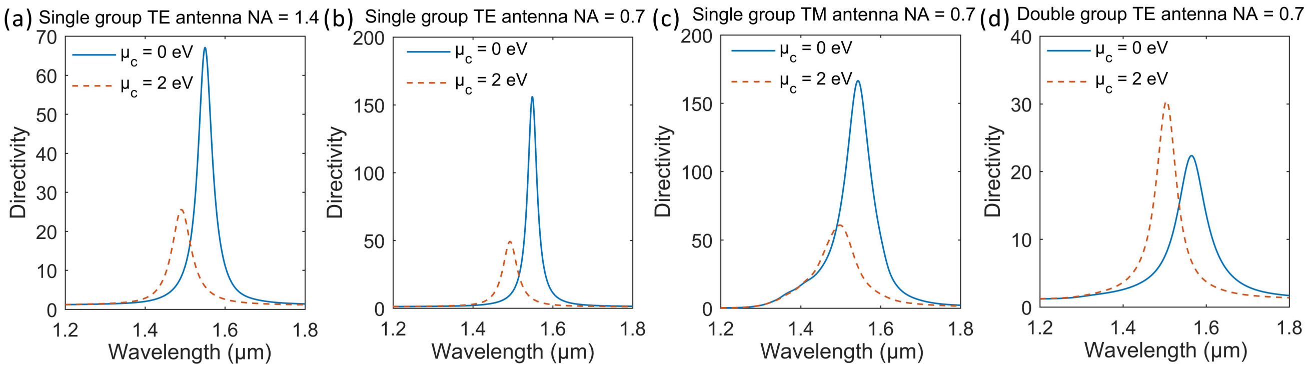

Fig. S 6. Tunability comparison under the illumination of focused Gaussian sources with different values of objective NA.

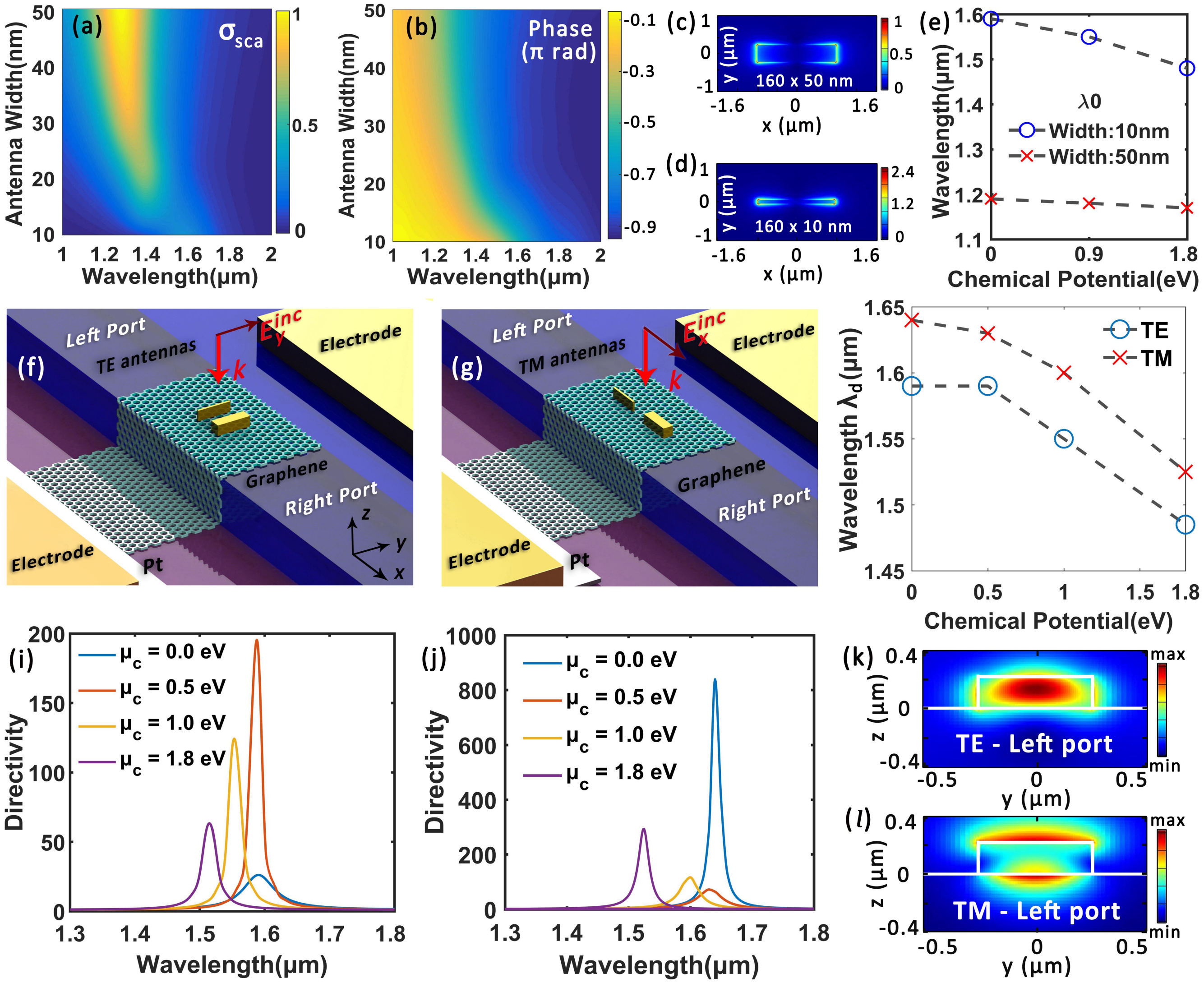

Fig. S 7. (a) and (b) Normalized scattering cross-section and phase response as a function of antenna width (fixed $l = 160$ nm $h = 50$ nm) under $\mu_c = 0.2$ eV. (c) and (d) $|\mathbf{E}|$ distributions on the antenna-graphene interface at resonant wavelength under $\mu_c = 0$ eV. (e) Antenna resonant wavelength versus μ_c for different antenna widths. (f) and (g) Device structures for directional couplings to left-propagating TE and TM mode respectively. The antenna center-to-center distances are 100 (f) and 280 nm (g). (h) Operation wavelength tunability analysis to (f) and (g). (i) and (j) Directivity curves under different μ_c for devices sketched in (f) and (g) respectively. (k) and (l) $|\mathbf{E}|$ distributions of left ports at 1.55 μm for (f) and (g) respectively.

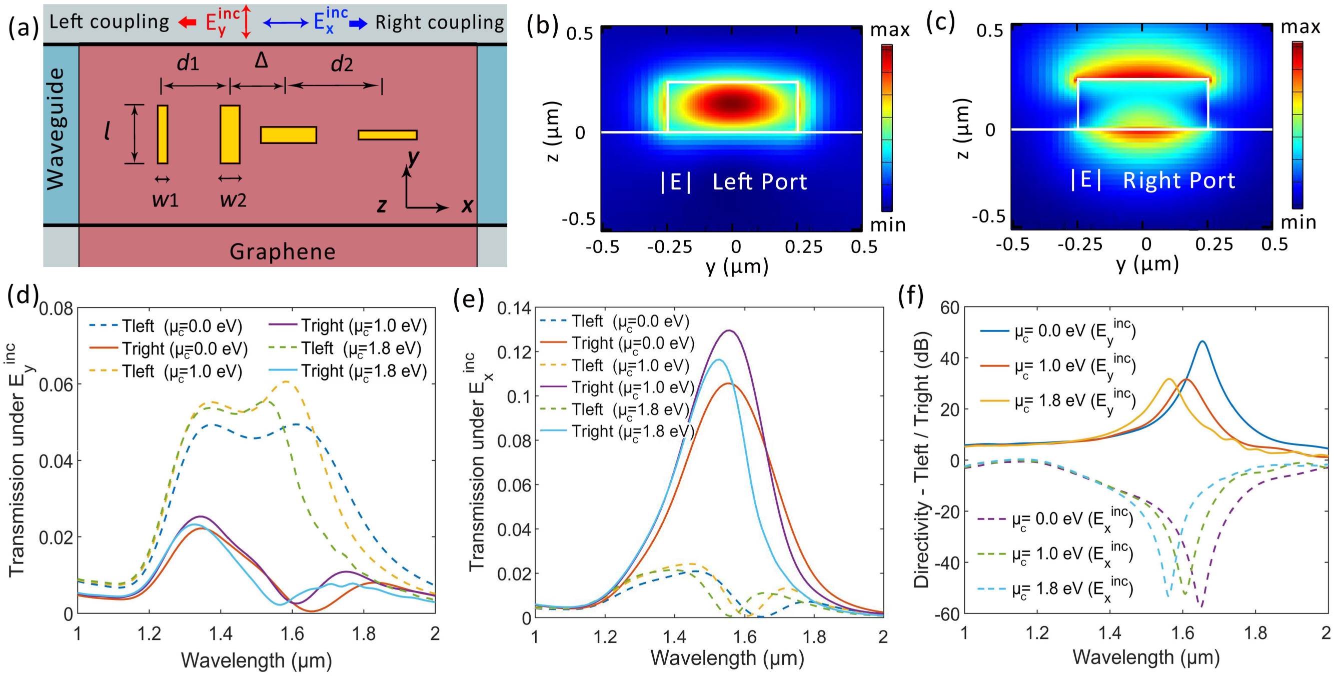

Fig. S 8. Proposal of an on-chip tunable polarization sorter. (a) Schematic sketch. Antenna element geometry (l, w_1 and w_2) is the same as Fig. S7(f) and (g). (b) and (c) The electric field norm distribution at the left and right waveguide ports respectively. (d) and (e) The transmission curve under different values of graphene's chemical potential μ_c under the linearly y- and x-polarized excitation light respectively. (f) Tunability analysis of the directional couplings.
